# An Automated Feature-Based Image Registration Strategy for Tool Condition Monitoring in CNC Machine Applications

**DOI:** 10.3390/s24237458

**Published:** 2024-11-22

**Authors:** Eden Lazar, Kristin S. Bennett, Andres Hurtado Carreon, Stephen C. Veldhuis

**Affiliations:** McMaster Manufacturing Research Institute (MMRI), Department of Mechanical Engineering, McMaster University, 230 Longwood Rd S, Hamilton, ON L8P0A6, Canada; bennetks@mcmaster.ca (K.S.B.); hurtada@mcmaster.ca (A.H.C.); veldhu@mcmaster.ca (S.C.V.)

**Keywords:** machine vision (MV), computer vision (CV), tool wear monitoring, image registration, condition monitoring, condition-based maintenance

## Abstract

The implementation of Machine Vision (MV) systems for Tool Condition Monitoring (TCM) plays a critical role in reducing the total cost of operation in manufacturing while expediting tool wear testing in research settings. However, conventional MV-TCM edge detection strategies process each image independently to infer edge positions, rendering them susceptible to inaccuracies when tool edges are compromised by material adhesion or chipping, resulting in imprecise wear measurements. In this study, an MV system is developed alongside an automated, feature-based image registration strategy to spatially align tool wear images, enabling a more consistent and accurate detection of tool edge position. The MV system was shown to be robust to the machining environment, versatile across both turning and milling machining centers and capable of reducing tool wear image capturing time up to 85% in reference to standard approaches. A comparison of feature detector-descriptor algorithms found SIFT, KAZE, and ORB to be the most suitable for MV-TCM registration, with KAZE presenting the highest accuracy and ORB being the most computationally efficient. The automated registration algorithm was shown to be efficient, performing registrations in 1.3 s on average and effective across a wide range of tool geometries and coating variations. The proposed tool reference line detection strategy, based on spatially aligned tool wear images, outperformed standard methods, resulting in average tool wear measurement errors of 2.5% and 4.5% in the turning and milling tests, respectively. Such a system allows machine tool operators to more efficiently capture cutting tool images while ensuring more reliable tool wear measurements.

## 1. Introduction

### 1.1. Background

With the advent of Industry 4.0, the ability to enable autonomous and self-adapting production through the integration of advanced digital and sensor technologies is critical to minimize production costs and enhance productivity [1]. This integration is particularly necessary in the context of Tool Condition Monitoring (TCM), which is often cited as a major source of inefficiency in the manufacturing process. These inefficiencies arise when machine cutting tools are replaced too frequently, leading to unnecessary machine downtime [2] or not frequently enough, resulting in unexpected tool breakage, inadequate product quality, and prolonged machine shutdown [3]. As a result, there remains a need to continuously improve TCM practices to monitor cutting tool health more efficiently and effectively during machining operations.

The process of TCM refers to the ongoing evaluation of cutting tool condition and performance during machining operations, encompassing both direct and indirect assessment methods. Traditionally, offline direct approaches utilize measurement systems such as digital and optical microscopes to evaluate tool wear directly, whereas offline indirect approaches inspect component dimensionality and surface texture to infer tool condition. Although offline direct TCM strategies provide more accurate and comprehensive assessments of tool conditions, their implementation is often too slow for practical use in production environments. Therefore, online TCM approaches have been developed to provide more efficient means to monitor tool conditions.

In the context of online indirect TCM strategies, such tasks are often performed through the implementation of cutting force, vibration, acoustic emission (AE), motor current, and temperature sensors [4]. Feature extraction is performed on acquired cutting signals in the time, frequency, or time-frequency domain and used as input for decision-making algorithms. These algorithms typically consist of artificial neural networks (ANNs), fuzzy logic (FL), adaptive neuro-fuzzy inference systems (ANFIS), hidden Markov models (HMMs), and support vector machines (SVMs), among others, to infer tool state conditions [5]. For instance, Segreto et al. developed a TCM multi-sensor strategy for the turning of Inconel 718 based on the acquisition of force, vibration, and acoustic emission signals using an ANN [6]. Time-frequency domain features were extracted using wavelet packet transform (WPT) and subsequently fed into an ANN for tool wear state condition estimation, yielding a mean absolute error percentage of 5.17%. Kuntoğlu et al. investigated the capacity for tool wear prediction and breakage detection using sensor fusion of the five aforementioned sensors during the turning of AISI 5140 [7]. The results indicated that while AE and temperature signals showed strong correlations with flank wear and enabled accurate predictions when integrated into an FL system, cutting force components exhibited weaker correlations and were unsuitable for similar applications.

While online indirect TCM strategies provide a means of monitoring tool conditions during machining, they are limited in their ability to detect and identify between particular wear mechanisms and failure modes, such as chipping, notching, and build-up edge (BUE) [8]. Moreover, these strategies demonstrate inconsistencies in the sensor data combinations that are effective for TCM. Furthermore, to develop the models necessary for tool wear state prediction, online indirect TCM strategies still rely on manually collected tool wear measurements, typically obtained through digital microscopes.

Online direct TCM strategies are primarily performed through the use of machine vision (MV) systems to extract tool wear measurements and classify cutting tool failure modes. While preliminary implementations of MV-TCM systems primarily focused on acquiring cutting tool images in situ and manually assessing tool condition, this practice has since evolved to incorporate more sophisticated image processing and machine learning methods to automate the process [9]. Feature extraction from tool wear images has been achieved through image processing techniques, including histogram transformation, segmentation, edge detection, and morphological operations, among others, to extract geometric, textural, and fractal features of the tool wear region. These features can be processed further to extract tool wear measurements using image processing alone or incorporated into machine learning models such as ANN, SVM, FL, and regression models, typically yielding more reliable and accurate tool condition assessments [8]. For instance, Wu et al. developed an MV system to automatically classify tool wear types based on a convolutional neural network (CNN) for the machining of Inconel 718 and demonstrated a high recognition precision rate of 96.20% [10]. This model was later used to detect tool flank wear measurements from acquired MV images and achieved a mean absolute percentage error of 4.76%. Ong et al. developed a framework for tool wear monitoring during the machining of high-speed steel using a wavelet neural network (WNN) [11]. Various combinations of input features, including cutting speed, feed rate, depth of cut, machining time, and the percentage of pixels corresponding to tool wear from processed cutting tool images, were used as input parameters in the WNN for predicting tool wear, achieving average percentage errors as low as 1.11%. However, the limitations of this setup included the manual acquisition of tool wear images using a digital microscope and the image processing being tailored to the specific wear pattern observed.

Direct TCM strategies provide highly accurate tool wear assessments and essential data for characterizing tool failure modes; however, integrating these strategies into online practices through MV systems remains challenging due to their sensitivity to the CNC machining environment, including factors such as physical mounting, lighting, incoming swarf and coolant [12]. Furthermore, a recent comprehensive review of MV systems in TCM applications found that a disproportionate number of these systems utilize advanced methods for tool wear extraction rather than relying solely on fundamental image processing techniques, demonstrating the complexity of automating this task [8].

A key challenge in MV-TCM image processing is the accurate extraction of the cutting tool edge position from each image. Precise edge detection is essential for establishing a reference line for tool wear measurement, particularly when the cutting edge is compromised by adhered material or chipping. This is especially critical given the diverse range of tool-workpiece and cutting parameter combinations used in manufacturing, which result in some conditions being more susceptible to material adhesion, such as BUE and welded-on chips, and others exhibiting progressive edge chipping.

The consistent and accurate assessment of the tool edge position is primarily influenced by two factors: (1) compounded mechanical and thermal positioning errors from the machine tool, coupled with MV mounting system instability, which result in positional deviations between images, and (2) the presence of BUE, welded-on chips, and edge chipping, which obstruct standard edge detection methods. These issues are often overlooked in the literature, as position variations in images can be managed through manual identification of the region of interest (ROI), while adhered material formation and chipping can be mitigated by strategic selection of tool-workpiece combinations and cutting conditions. However, such approaches undermine the robustness and reliability of the MV-TCM system. For instance, Mikołajczyk et al. employed a single category-based classifier neural network for automatic tool wear analysis, achieving an absolute mean relative error of 6.7% [13]. However, this framework depended on manual segmentation of the tool wear region, rendering it susceptible to variations in wear patterns. Additionally, the study did not account for conditions of material adhesion or edge chipping. Peng et al. developed an MV-TCM system within a CNC milling center, employing image processing to detect tool wear conditions and predict flank wear measurements with a mean error of 5.73% [14]. However, the framework did not address the detection and differentiation of adhered material formation and relied on a fixed, pre-specified ROI for tool wear analysis. Furthermore, the MV camera and ring light assembly were directly exposed to the machining environment, rendering the system susceptible to damage over time. Hrechuck et al. developed a framework to automate the characterization of tool wear morphology with the added ability to distinguish between tool wear and adhered material formation [15]. However, a limitation of this work was the reliance on an aggregate channel feature (ACF) object detector trained over several hundred labeled tool wear images to perform the tool wear assessment. Sun et al. integrated an MV system within a CNC turning center to classify the status of cutting tools, distinguishing among flank wear, chipping, fracture, and BUE conditions [16]. The MV system was housed in a compact protective enclosure and mounted to the back panel of the CNC machine; however, noticeable deviations in perspective were observed between tool wear images. These discrepancies were likely attributed to transmitted vibrations and errors in machine tool positioning repeatability throughout the image acquisition sequence. To address this issue, Hough transforms were employed under varying lighting conditions to detect the contour lines of the cutting tool and flank face. The identified reference lines were then used to assess cutting tool states based on the spatial relationships of detected features to the contour lines. While this method demonstrated effectiveness in broad tool state classification, its reliability for measuring low levels of tool wear (*Vb_av_* < 100 μm) was not investigated.

While advancements in MV-TCM systems have enabled automated tool wear assessment and classification, challenges such as positional deviations, material adhesion, and edge chipping continue to undermine system robustness and reliability.

### 1.2. Image Registration Methods for MV-TCM Systems

Image registration refers to the process of spatially aligning two or more image sets taken of the same scene but from different viewpoints, times, or sensor modalities [17]. This technique is widely applied in fields such as medical imaging [18] and remote sensing [19]; however, its application within MV-TCM systems remains limited.

Liang et al. were among the first to employ image registration techniques to monitor the progression of tool nose wear in carbide milling inserts [20]. Contour images of the cutting tool were captured and segmented into sub-areas to extract vertex features of the tool edge. These features were subsequently matched using calculations of marginal entropy, joint entropy, and mutual information. The differences in tool edge contours between the unworn and worn states were utilized to evaluate the degree of tool wear. Ramzi et al. investigated the progression of flank wear in drilling applications by employing image registration to compare the drill tip geometry between worn and unworn states [21]. However, it was noted that the registration process proved effective only at low stages of tool wear, leading to increasingly inaccurate transformations as the wear progressed. Consequently, scale and rotation coefficients were manually adjusted during subsequent registrations to maintain accurate image alignments. Malhotra et al. developed a c-means clustering-based image segmentation technique for monitoring tool wear in micro milling, incorporating an intensity-based image registration sequence to align wear images prior to analysis [22]. The registration process was found to be critical for background removal and ROI extraction, achieved through a histogram projection-based segmentation technique. However, tool wear measurements were sensitive to contaminants and adhered material formation, which could not be effectively detected through ROI extraction or eliminated using air jet cleaners, therefore requiring manual cleaning before image acquisition. Furthermore, the optimization sequence inherent in intensity-based registrations often demands substantial computational resources, which may limit the applicability of this technique in online TCM applications. Zhou et al. developed a chisel edge wear measurement system for high-speed steel twist drills utilizing a projection-based image registration method [23]. This framework enabled efficient ROI extraction; however, its accuracy was dependent upon the quality of the initial edge detection, which would be sensitive to factors such as lighting conditions and threshold selection. Additionally, the system required the removal of the cutting tool for image acquisition, limiting its applicability for online monitoring. Dou et al. implemented a coherent point drift (CPD) registration algorithm to align tool wear images prior to rake wear detection in endmills [24]. However, excessive noise present on the cutting tool face necessitated the use of a fixed reference indicator within the frame to ensure accurate registration.

### 1.3. Research Gaps and Contributions

The review of the literature presented above emphasizes the need for a more robust strategy for edge detection, particularly in cases when the tool edge is compromised by adhered material or chipping. While advanced processing techniques have been employed to address these challenges, the underlying issue lies in the relative positional variations between the MV system and the cutting tool in each image caused by machine tool positioning errors and the instability of the MV system. Prioritizing spatial alignment of tool wear images presents a more effective solution compared to current methods; however, the implementation of registration techniques in MV-TCM remains limited. Current studies predominantly utilize fixed registration frameworks despite the increasing difficulty in achieving accurate transformations as tool wear progresses. They also often perform registrations under a single tool coating condition, leaving the performance of these techniques across varying coatings unexplored. Further, they have yet to implement standard feature-based registration techniques despite their accessibility and extensive use in the literature. Finally, while automating the registration process would be essential for an online MV-TCM strategy, it has not yet been explored in the current literature.

The research gaps in direct TCM applications, particularly concerning the implementation of MV systems for online monitoring, can be summarized as follows:

1. Standard TCM image acquisition practices are time-intensive and interfere with production cycles.
2. MV designs are sensitive to the machining environment, including vibrations, swarf, coolant, and lighting variations, requiring continuous oversight and maintenance.
3. Machine tool positioning errors and MV system instability during image acquisition are often overlooked in MV-TCM, complicating the tool edge detection process and increasing measurement inaccuracies.
4. Registration practices in MV-TCM utilize fixed registration frameworks, operate under single coating conditions, and have not been automated for online practices.

These drawbacks limit the effective implementation of MV systems in TCM, resulting in the need for manual collection of cutting tool images and wear measurements. To address these challenges, this study establishes three key objectives: (1) to develop an MV system that is robust to the machining environment, adaptable across various CNC machine centers, and capable of capturing high-quality tool wear images; (2) to develop an automated registration algorithm tailored for MV-TCM applications; and (3) to establish a more effective tool edge detection strategy based on spatially aligned tool wear images.

The remaining sections of this article detail the study’s research. Section 2 presents the MV system design and experimental setup. Section 3 covers system validation, the development of an automated feature-based image registration algorithm, and the registration-based reference line detection strategy for MV-TCM. Section 4 provides a case study demonstrating the registration-based reference line detection strategy’s application in a CNC milling center. Finally, Section 5 summarizes the findings and discusses directions for future research.

## 2. Materials and Methods

### 2.1. Proposed Framework

**Figure 1:**
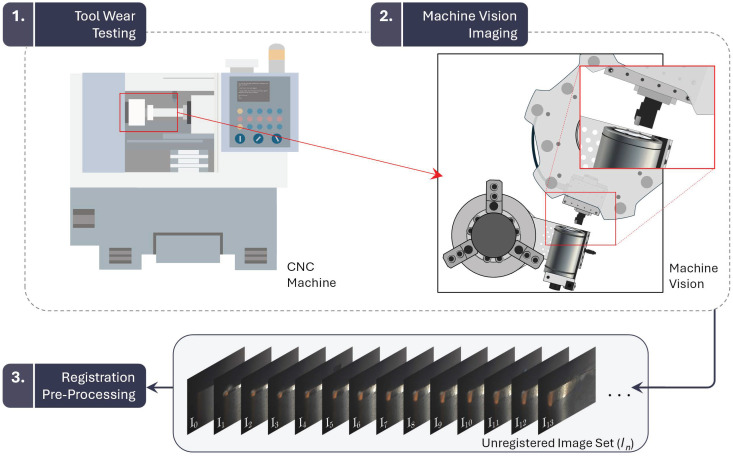
Image acquisition framework of the MV-TCM system.

Figure 1 presents the image acquisition framework, outlining the two principal steps of a standard MV-TCM process: (1) tool wear testing and (2) MV imaging. It also introduces a third step, a registration-based pre-processing stage, which performs the spatial alignment of images prior to the tool wear analysis.

The research program was divided into three stages. In the first stage, the MV system was integrated into a CNC lathe to capture tool wear images following a series of tool life-cutting tests, which served to validate the system. In the second stage, an automated registration algorithm was developed, informed by a comparative performance analysis of five feature detector-descriptor algorithms alongside evaluations of challenging registration cases, coating variation effects, and the impact of different registration frameworks. In the third stage, the algorithm was adapted to a registration-based reference line detection strategy for tool wear assessment and validated for CNC turning applications. A case study is later presented, demonstrating the application of the registration-based MV-TCM strategy within a CNC milling center.

The image acquisition framework should enable the efficient collection of cutting tool images without the need to remove the tool from the CNC machine, while the registration-based reference line detection strategy should simplify the process of tool edge detection to provide a more consistent and accurate assessment of tool wear.

### 2.2. MV-TCM Hardware and Software Systems

#### 2.2.1. MV System Design

**Figure 2:**
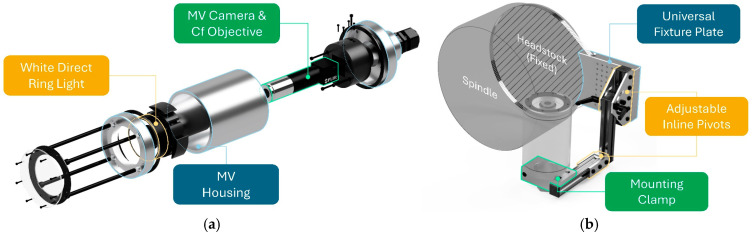
Schematic of the MV design showcasing the (**a**) housing and (**b**) mounting components. Mounting is configured for a turning center, oriented to capture the primary flank face of the tool.

The MV apparatus, shown in Figure 2, was designed as a two-part system: (1) the housing, used to enclose critical imaging components, and (2) the mounting, used to support and articulate the MV system.

The housing system, shown in Figure 2a, consists of four components, including (1) the MV camera, (2) the objective lens, (3) the illumination system, and (4) the protective enclosure. For this setup, the BFS-U3-50S5C-C ultra-compact FLIR MV camera was selected, along with the #88-353 Edmund Optics CF objective lens and CA-DRW7 KEYENCE white direct ring light. The enclosure was designed as a three-piece assembly made of Al-6061 tubing. The front component supports a scratch-resistant, exchangeable cast acrylic sheet, the middle section provides clearance and support for the lens and ring light, and the back piece supports the MV camera while serving as a fixing point for the mounting system. Oil-resistant Buna-N gaskets were embedded between contacting components, and a cable gland was incorporated to ensure resistance to machining cutting fluids. To maximize rigidity, custom-printed supports were positioned between the MV camera, objective lens, and ring light, securing them to the inner wall of the enclosure. Finally, the system was firmly secured using four internal bolts.

The mounting system, shown in Figure 2b, consists of three components, including (1) the universal fixture plate, (2) T-slotted framing pivots, and (3) the stanchion mounting clamp. The fixture plate served as the primary mounting source for the MV system and was secured against the CNC machine headstock using a hose clamp. Inline pivot arms were incorporated to enable positional control of the MV system relative to the cutting tool. Finally, a stanchion mounting clamp was implemented, enabling a rigid connection to the MV housing system and allowing full 360° rotational control of the MV camera. The design of the mounting system allows for further reconfiguration, enabling image acquisition of the secondary flank face, rake face, and various perspectives in between. Reconfiguration into CNC milling centers is also feasible and demonstrated in Section 4.

#### 2.2.2. MV Software & Registration Functions

In this article, the performance of five feature detector-descriptor algorithms was assessed using collected MV images. The algorithms included the Scale Invariant Feature Transform (SIFT), Speeded-Up Robust Features (SURF), KAZE, Binary Robust Invariant Scalable Keypoints (BRISK), and Oriented FAST and Rotated BRIEF (ORB). Detector-descriptor parameters were selected based on literature recommendations and remained consistent throughout testing. All processing tasks were executed in MATLAB 2022b, utilizing the Image Processing Toolbox™ (Version 11.6) on a computer system equipped with an Intel^®^ Core i7-4770HQ CPU (2.20 GHz, 6 MB Cache) and 16.00 GB of RAM.

Table 1 summarizes the five feature detector-descriptor algorithms employed in this work, along with their corresponding MATLAB functions, input parameters selected, and general properties. Additional details regarding the formulation of these algorithms and their applications are provided in Section 2.2.3.

The registration process was initiated with feature detection, where distinct features were identified in the target and reference images by applying a feature detector function. For each feature, a descriptor was computed by applying its corresponding descriptor function to provide a unique characterization of the feature. The feature-matching process was performed by computing pairwise distances between descriptor vectors to identify corresponding features between the two images. For non-binary descriptors, the sum of squared differences (SSD) was utilized, while the Hamming distance was employed for binary feature vectors. To detect the highest possible number of matches, the match threshold ($T_{m}$), a scalar representing the distance from a perfect match between two descriptor vectors, was set to the maximum value of 100. The ratio threshold ($T_{R}$), a parameter designed to eliminate ambiguous feature matches by comparing the distances of the closest and second-closest matches for each pair, was set to the default value of 0.6.

While most transformations produced from the matching process represented true responses between correctly matched pairs, a small portion of erroneous transformations were generated from false positive matches. The M-estimator Sample Consensus (MSAC) algorithm was implemented to refine the matches by iteratively identifying and removing outliers. The algorithm was configured with 1000 trials, a 99% confidence interval, and a maximum distance threshold of 1.5 pixels, representing the allowable deviation for a transformed point from its true projected location. Finally, the transformation matrix was computed using the remaining inlier matches. In this work, a two-dimensional similarity transformation, shown in Equation (1), was employed to perform the registrations:

(1) $$\left[\begin{array}{c} x^{\prime }\\ y^{\prime }\\ 1 \end{array}\right]=\left[\begin{array}{ccc} \alpha_{t}\cos\left(\theta_{t}\right) & -\alpha_{t}\sin\left(\theta_{t}\right) & t_{x}\\ \alpha_{t}\sin\left(\theta_{t}\right) & \alpha_{t}\cos\left(\theta_{t}\right) & t_{y}\\ 0 & 0 & 1 \end{array}\right]\left[\begin{array}{c} x\\ y\\ 1 \end{array}\right],$$

where ($x^{\prime },y^{\prime }$) and (x,y) are coordinates of the reference and target image respectively, ($\alpha_{t}$) represents the uniform scale factor, ($\theta_{t}$) is the rotation angle, and ($t_{x}$) and ($t_{y}$) represent the horizontal and vertical displacements, respectively. In this work, two registration frameworks were utilized: a fixed framework, where the reference image remained constant throughout the process, and a sequential framework, where the reference image was updated to the most recently registered target image.

Unsuccessful image registrations, characterized by visibly skewed final transformations, were reattempted using relaxed ratio thresholds within the range of 0.6 to 0.8, typically utilized in the literature, until an accurate transformation was achieved. Image registrations that were unable to produce accurate alignments despite the relaxed ratio thresholds were classified as failed registrations. The performance of the feature detector-descriptor algorithms was evaluated based on five metrics: (1) average number of features detected; (2) average number of matched feature pairs detected; (3) ratio of inlier features to detected features; (4) computational time; and (5) number of failed registrations.

#### 2.2.3. Feature Detector-Descriptor Algorithms

The SIFT algorithm, introduced by D.G. Lowe in 2004 [25], operates by applying the Difference-of-Gaussian (DoG) operator to images represented in various scales within the scale-space domain. Features, or keypoints, are detected across scales by identifying local minima and maxima and are discarded if they do not meet contrast or localization thresholds. Equation (2) shows the convolution of the DoG at different scales:

(2) $$D\left(x,y,\sigma \right)=L\left(x,y,k\sigma \right)-L\left(x,y,\sigma \right),$$

where $L\left(x,y,\sigma \right)$ represents the scale-space of an image, σ is the scale, and k is a multiplicative factor. The SIFT descriptor is represented by a 128-dimensional vector, formulated by extracting 16 × 16 neighborhoods around each keypoint, further dividing the region into 4 × 4 blocks, and computing 8-bin orientation histograms for each block. The algorithm has been widely used for optical image registrations and has recently been adapted successfully for synthetic aperture radar (SAR)-optical image registration applications—a challenging task due to differences between imaging modalities, variations in image intensity values, and the presence of strong speckle noise and complex local distortions in SAR images [30]. Comparative analyses of feature detector-descriptor algorithms consistently demonstrate that SIFT offers superior accuracy and repeatability, although it tends to have somewhat lower computational efficiency [31,32,33].

The SURF algorithm, presented by H. Bay et al. in 2008 [26], operates closely to SIFT, relying on scale-space analysis of the image; however, it improves computation speed by using integral images to approximate the Hessian matrix for feature detection. Equation (3) shows the Hessian of an image at point $X=\left(x,y\right)$:

(3) $$H\left(X,\sigma \right)=\left[\begin{array}{cc} L_{xx}\left(X,\sigma \right) & L_{xy}\left(X,\sigma \right)\\ L_{xy}\left(X,\sigma \right) & L_{yy}\left(X,\sigma \right) \end{array}\right],$$

where $L_{xx}\left(X,\sigma \right)$, $L_{yy}\left(X,\sigma \right)$, and $L_{xy}\left(X,\sigma \right)$ represent the second-order partial derivatives of the scale-space image with respect to point X. The SURF descriptor is represented by a 64-dimensional vector, formulated by computing Haar wavelet responses in the horizontal and vertical direction relative to the main orientation of the feature descriptor, within 4 × 4 blocks around each keypoint. The SURF algorithm has also been extended to remote sensing applications due to its computational efficiency and robustness to lighting variability [34]. Comparative analyses generally demonstrate a balanced performance in terms of accuracy and overall efficiency for the SURF algorithm [31,32,33].

The KAZE algorithm, introduced by P.F. Alcantarilla et al. in 2012 [27], operates by detecting keypoints using nonlinear scale-space analysis. This method facilitates adaptive blurring of images, which preserves edge details while reducing noise to enhance localization accuracy and feature distinctiveness. Equation (4) illustrates the nonlinear diffusion expression used to achieve local adaptive blurring of images:

(4) $$\frac{\partial L}{\partial t}=div\left(c\left(x,y,t\right)\cdot \nabla L\right),$$

where div represents the divergence operator and ∇ represents the gradient operator. The conductivity function c is defined as shown in Equation (5):

(5) $$c\left(x,y,t\right)=g\left(\left|\nabla L_{\sigma }\left(x,y,t\right)\right|\right),$$

where $\nabla L_{\sigma }$ represents the Gaussian smoothed version of the original image. Feature detection in KAZE relies on the scale-normalized determinant of the Hessian matrix, where keypoints are identified as maxima in the response. Rotational invariance is achieved similarly to SURF by computing the dominant orientation within a 6σ radius around each keypoint; however, the summed responses of first-order derivatives in the horizontal and vertical directions are used instead. The descriptor is then formulated by assessing these derivative responses relative to the main orientation within a 4 × 4 grid region, resulting in a 64-dimensional vector. The KAZE algorithm has been widely used in remote sensing applications, proving especially effective in SAR image matching due to its capacity to handle nonlinear speckle noise inherent in SAR imagery [35]. However, its reliance on non-diffusion filtering often results in longer computation times [31,32,33]. To address this, various modifications have been proposed to reduce computation time [36] or enhance matching efficiency [37]. Notably, Liu et al. achieved a significant improvement in image-matching performance by pairing KAZE features with SIFT descriptors [38].

The BRISK algorithm, introduced by S. Leutenegger in 2011 [28], operates by applying the Adaptive Generic Corner Detection Based on the Accelerated Segment Test (AGAST) algorithm in the scale-space to compute feature saliency scores. The BRISK descriptor is represented by a 64-dimensional binary vector, formulated from a pattern-based sampling of the neighborhood around the keypoint, with brightness comparison tests used to generate the descriptor. While BRISK has been employed less frequently in remote sensing registration applications compared to other methods, it has demonstrated performance comparable to SIFT, with the advantage of reduced computational time [39]. Additionally, extensions of the BRISK algorithm have been developed, further enhancing registration accuracy while reducing computational time, and have shown to be effective in orthoimage registration [40]. Comparative analyses of feature detector-descriptor algorithms consistently show that BRISK offers a favorable balance among accuracy, repeatability, and computational efficiency compared to other methods [31,32,33].

The ORB algorithm, introduced by E. Rublee in 2011 [29], uses a modified Features from Accelerated Segment Test (FAST) keypoint detector [41] applied across various scale-space representations of the image while using Harris Corner scores as a measure for feature saliency. The ORB descriptor is represented by a 32-dimensional binary vector formulated using a direction-normalized version of Binary Robust Independent Elementary Features (BRIEF) [42]. Enhancements to the ORB algorithm have been implemented in remote sensing image registration applications, including the integration of adaptive corner detection thresholds to increase feature point detection [43] and the use of a three-patch pixel comparison method to reduce descriptor sensitivity to noise [44]. However, comparative analyses between various feature detector-descriptors have consistently demonstrated that while the ORB algorithm offers superior computational efficiency, it exhibits reduced accuracy and repeatability [31,32,33].

### 2.3. Testing Methods

#### 2.3.1. MV System Validation

To evaluate the performance of the MV system, a comparative analysis was conducted against the standard manual TCM procedure within a CNC turning center. The comparison focused on two key aspects: (1) the overall quality and interpretability of the captured images and (2) the efficiency of the TCM process.

**Figure 3:**
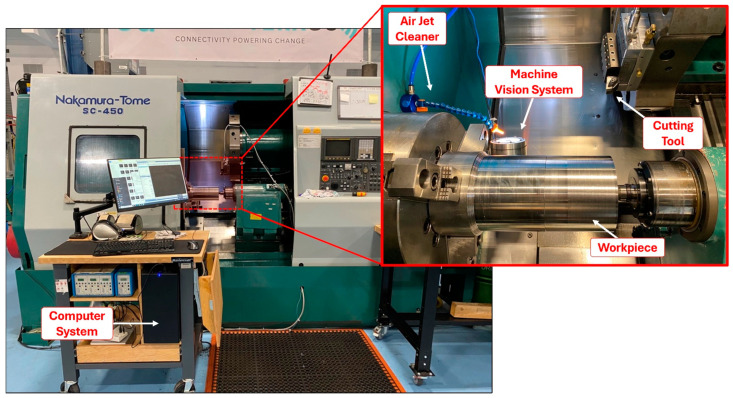
MV-TCM experimental setup for the CNC turning center.

The MV system was mounted on the CNC lathe headstock and oriented upwards to capture tool wear images of the primary flank face. The CNC machining center used was a Nakamura-Tome SC-450 (Hamilton, ON, Canada), equipped with a DCLNL 164CKC3 Kennametal tool holder. Uncoated carbide K313 inserts (0.8 mm nose radius, 30 μm edge radius) were utilized for tool wear tests. The selected workpiece material was AISI 316 stainless steel, measuring 304.8 mm in length and 150.4 mm in diameter. Figure 3 illustrates the experimental setup used in this work.

A factorial design of tool life-cutting tests was performed, consisting of three levels of cutting speed, two levels of feed rate, and a constant depth of cut, as outlined in Table 2. Tool wear tests were repeated twice, producing a dataset of 12 cutting tests. The experiments were conducted in a randomized order, corresponding to the MV dataset numbers.

Following each cutting pass, the CNC machine spindle rotation was suspended, a tool changeover was prompted, and the tool was repositioned ahead of the MV system. A delay period of 10 s was set to allow machine vibrations to decay, after which a sequence of four images, spaced 1 s apart, were captured and saved in the TIFF image file format with LZW lossless compression. The cutting tool insert was removed from the machine tool holder and positioned under the digital microscope. Tool wear imaging and flank wear measurements ($Vb_{av}$ and $Vb_{max}$) were conducted manually using the KEYENCE VHX-950F digital microscope, with measurements averaged from assessments by two independent experts. These measurements provided the reference values for subsequent MV-TCM evaluations produced in Section 3.3.2. The VH-Z20R lens, equipped with full ring light illumination, captured tool wear images at 200× magnification. Focus stacking, utilizing the depth composition feature, was employed to achieve fully focused images of the cutting tool. This procedure was repeated until the tool life criterion of 300 μm was reached, in accordance with ISO 3685:1993 [45].

#### 2.3.2. MV System Milling Case Study Setup

To assess the performance and broader applicability of the MV-TCM strategy, a case study was conducted by integrating the MV system within the QUICKMILL Eliminator HD 5A CNC milling center. Cutting tests utilized uncoated, 16 mm diameter, two-flute, ball-end milling inserts (DIJET BNM-160) along with a corresponding tool holder (DIJET BNMS-160033S-dS16C). A factorial design of cutting tests was performed, featuring two variations of workpiece materials and two levels of spindle speed, as outlined in Table 3. The experiments were conducted following the order of the MV dataset numbering, with odd and even dataset numbers corresponding to the first and second cutting tool edges.

The workpiece consisted of two variations of mold steel, each with dimensions 185.7 × 260.0 × 63.5 mm. Face milling operations were performed using a tilt angle of 25° relative to the feed direction, under flood coolant conditions, with a splash guard in place to prevent coolant interference during image acquisition. The cutting tool was intermittently repositioned ahead of the MV system to capture an image of both cutting edges and saved following the same procedure used in the turning experiments. The cutting tool would be removed and positioned under the digital microscopes for tool wear imaging and flank wear measurements, following the same procedures listed previously.

## 3. Results & Discussion

### 3.1. Validation of the MV System

**Figure 4:**
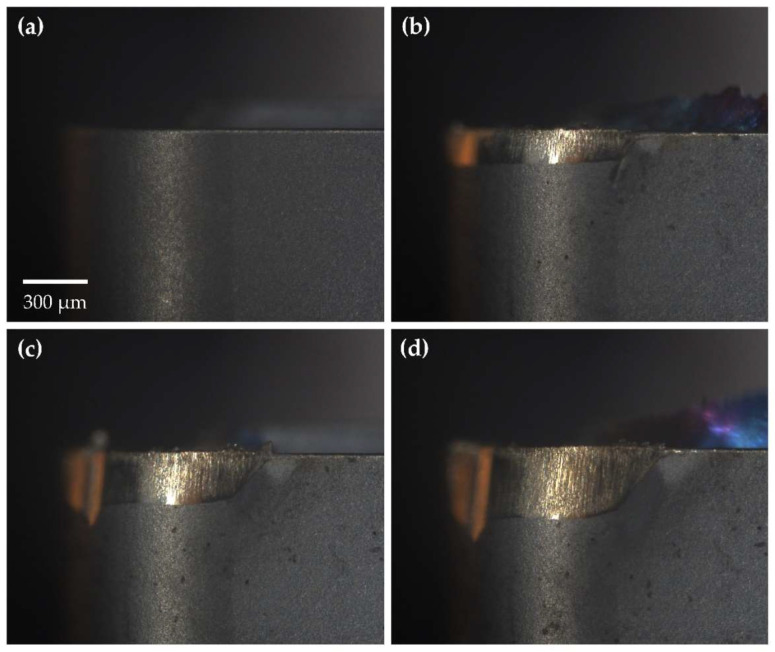
Tool wear images captured by the MV system (Image size: 2448 × 2048 pixels). Images collected from the T_3_ MV dataset at cutting pass: (**a**) P = 0; (**b**) P = 3; (**c**) P = 6; and (**d**) P = 9.

**Figure 5:**
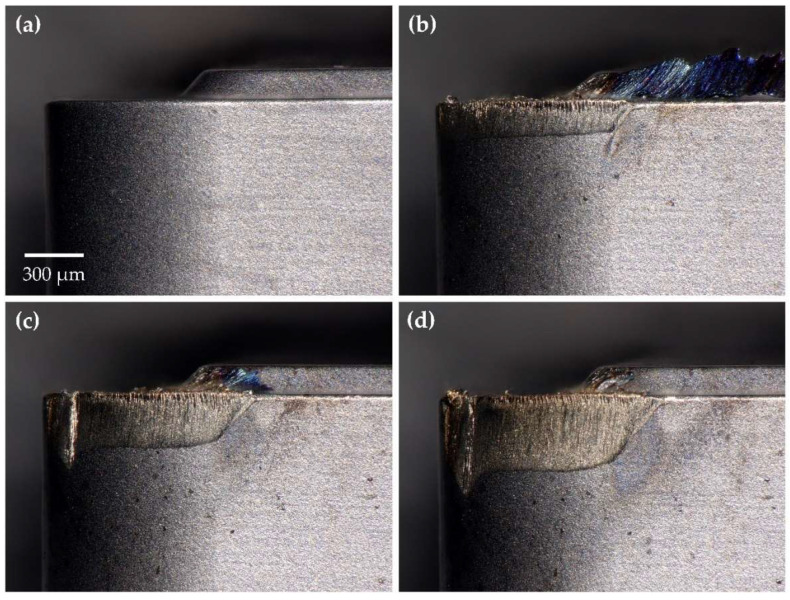
Tool wear images captured by the KEYENCE digital microscope (Image size: 1600 × 1200 pixels). Images collected from the T_3_ dataset at pass: (**a**) P = 0; (**b**) P = 3; (**c**) P = 6; and (**d**) P = 9.

Cutting tool wear tests were conducted as outlined in Section 2.3.1, producing 239 tool wear images, including both worn and unworn states of the tool, under six distinct cutting conditions. Figure 4 illustrates the progression of tool wear at various stages as recorded by the MV system while corresponding images captured using the KEYENCE digital microscope are presented in Figure 5 for comparison.

Images acquired with the digital microscope were generally brighter and exhibited higher spatial resolution than those captured by the MV system. The KEYENCE digital microscope employed focus stacking, effectively increasing the depth of field (DOF) and enhancing the visibility of cutting tool surface details at varying depths. Despite these differences, the MV system produced high-quality images with visible surface features and clearly distinguishable wear regions. The MV system’s field of view (FOV) also closely matched that of the digital microscope, further validating its suitability for tool wear assessment. With a DOF of 12 μm, the MV system adequately resolved surface details on the primary flank face without the need for additional processing. However, finer surface details along the tool nose radius and welded-on chips near the rake face were occasionally out of focus, depending on their distance from the focal plane. While these features offer a more comprehensive view of tool wear mechanisms and wear morphology, they are not critical for assessing the degree of tool wear. Therefore, this limitation was deemed acceptable for the purposes of this study.

The relative positioning between the cutting tool and MV system remained consistent throughout the testing process; however, minor positioning deviations were observed between successive images. These deviations were maintained within a ±50 μm displacement in both horizontal and vertical directions, as further demonstrated in Section 3.2.1. This level of variance is well within the scale at which tool wear is measured (300 μm, $Vb_{av}$) and the dimensions of the captured image (1427 × 1706 μm).

Vibrations transmitted from the CNC machine to the cantilevered mounting system, along with compounded geometric, kinematic, and thermal errors inherent to the CNC system, were suspected to be the primary contributors to the observed positional deviations. However, the underlying mechanisms driving these errors remain to be fully explored. While reducing the mounting system’s degrees of freedom could mitigate the observed positional variations, such modifications could compromise image quality by limiting the system’s positional control. Furthermore, machine tool errors are largely unavoidable and tend to become more pronounced with increased tool travel paths and higher imaging magnifications. Therefore, despite the MV system’s ability to provide stable imaging throughout tests, the use of image registration processing remains necessary to achieve precise spatial alignment of the cutting tool wear images.

To assess the efficiency of the image acquisition process between the MV system and the conventional manual method, a time study was conducted in parallel with the cutting tests. The TCM procedure was divided into three tasks: (1) Machining, (2) Setup/Removal, and (3) Image Capture. A breakdown of the steps involved in each task for both methods is provided in Table 4. The machining task, which included the time required to complete all cutting passes, was consistent between both methods and excluded from the table.

Table 5 summarizes the results of the time study analysis comparing standard and MV-TCM methods. Tool life test durations decreased progressively across test conditions due to the increased cutting speeds and feed rates.

**Figure 6:**
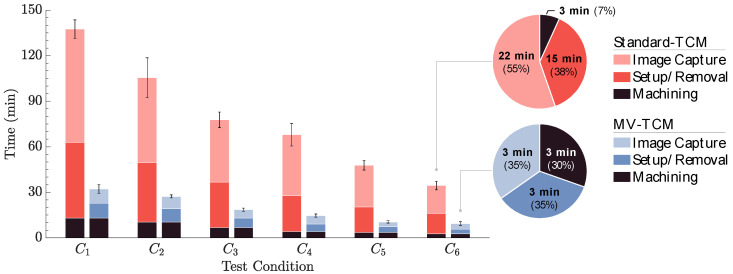
Time study comparison between standard and MV-TCM systems. The bar chart columns are ordered by test condition corresponding to cutting tests: T_7_, T_4_, T_1_, T_2_, T_10_, and T_3_, respectively.

The MV system demonstrated a significant improvement in efficiency, reducing the image acquisition time by 84% on average across all tests compared to the standard manual procedure. The analysis further highlights a distinct contrast between the two methods in terms of time distribution across tasks, as shown in Figure 6. In the standard-TCM setup, machining accounted for 8% of the total time, while setup/removal and image capture consumed 37% and 55%, respectively. In contrast, the MV-TCM setup achieved a more balanced time allocation across all tasks.

The time study comparison highlights the MV-TCM system’s capacity to reduce process variability by minimizing operator intervention. Beyond the time savings, this reduction in variability enhances machining consistency by shortening intervals between cutting passes and promotes operator safety by limiting direct interactions with the machine, thereby reducing the likelihood of human error. Further time reductions may be achieved by implementing a trigger timing controller to automate image acquisition.

### 3.2. Feature-Based MV-TCM Registration

#### 3.2.1. Performance Case Study of Feature Detector-Descriptor Algorithms

The registration of acquired MV images was conducted following the procedures outlined in Section 2.2.2. To focus the analysis solely on evaluating the performance of the feature detector-descriptors, registrations were performed sequentially, with each aligned image serving as the reference image for the subsequent registration. The validity of the registration was assessed by visually comparing the registered target image against the previous reference image. The performance results of the five feature detector-descriptor algorithms are summarized in Table 6.

Among the tested algorithms, SIFT offered a good balance between registration accuracy and efficiency, yielding 2/227 (0.88%) failed registrations, with an average computational time of 2.90 s per registration. While comparatively detecting fewer features on average, the SIFT algorithm demonstrated the highest inlier-to-detected (ITD) feature ratio of 1:6, described as the average number of feature pairs required to detect a single inlier feature pair. A higher ITD ratio is indicative of a more robust feature detector-descriptor algorithm, primarily due to its ability to detect similar features across both reference and target images, coupled with highly differentiable feature descriptors, resulting in significantly fewer false-positive matches.

The SURF algorithm offered a comparatively greater computational efficiency, with an average time of 0.91 s per registration, but at the cost of registration accuracy and repeatability, yielding 14/226 (6.17%) failed registrations. The algorithm further detected the fewest number of features and produced a comparatively lower ITD ratio of 1:19.

The KAZE algorithm offered superior registration accuracy, yielding 1/227 (0.44%) failed registrations but at the expense of computational efficiency, with an average computational time of 9.89 s per registration. Furthermore, KAZE produced the highest number of detected features along with the highest ITD feature ratio (1:6), highlighting its potential implementation for MV-TCM registration applications.

The BRISK algorithm offered a more balanced performance between registration accuracy and efficiency, yielding 6/227 (2.64%) failed registrations, with an average computational time of 2.55 s per registration. The algorithm was shown to detect a comparatively high number of features but produced a lower ITD feature ratio of 1:25.

Finally, the ORB algorithm offered a superior balance between registration accuracy and efficiency, yielding 3/227 (1.32%) failed registrations, with an average computational time of 0.50 s per registration. Although the algorithm was shown to detect a comparatively high number of features, it exhibited the lowest ITD feature ratio of 1:30. This ratio was driven by the high number of outlier-matched feature pairs, indicating challenges in identifying similar features across images and distinguishing true correspondences from false matches, leading to more false positives. Despite this, the algorithm performed effectively when a sufficient number of features were present across the images, with its greatest advantage being its computational efficiency, making it well-suited for rapid registrations in MV-TCM applications.

Table 7 summarizes the comparative performance of the five feature detector-descriptor algorithms based on two measures: (1) Overall Accuracy, which reflects the algorithm’s ability to achieve successful registrations, evaluated by the number of registration errors produced, and (2) Overall Efficiency, which represents the computational time required to complete the registration process. Performance results are presented on a relative scale from one to five stars, with higher ratings indicating superior performance.

Detected features were primarily distributed across regions of high-contrast areas, including the wear region and along the tool edge. Matched feature pairs were predominantly observed in the brighter, unworn regions of the tool surface, irrespective of the feature detector-descriptor employed. A notable reduction in inlier feature pairs was observed in the worn regions, likely due to textural changes from machining, which hindered the feature-based algorithms’ ability to consistently identify matches in these areas.

**Figure 7:**
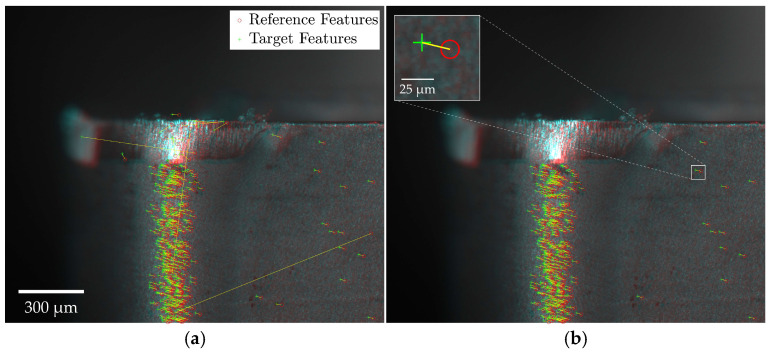
MV-TCM registration using the T_8_ dataset, (*I_ref._* = I_6_) & (*I_tgt._* = I_7_) and employing the SIFT algorithm (*T_R_* = 0.6). Registration depicted using overlayed images (**a**) including all matched feature pairs and (**b**) following outlier removal using MSAC algorithm.

Figure 7 provides a schematic of the feature matching and outlier removal process. The registration outcomes are visualized by overlaying reference and target images, with discrepancies indicated in red and cyan. Specifically, red areas represent bright regions in the target image absent from the reference, while cyan areas represent bright regions in the reference image not found in the target. Features detected in the reference and target images are marked by a red circle and green cross, respectively, with a yellow line indicating the transformation vector required to align matched feature pairs. Figure 7a displays all potentially matched feature pairs, while Figure 7b highlights the inlier pairs retained after outlier exclusion using the MSAC algorithm.

**Figure 8:**
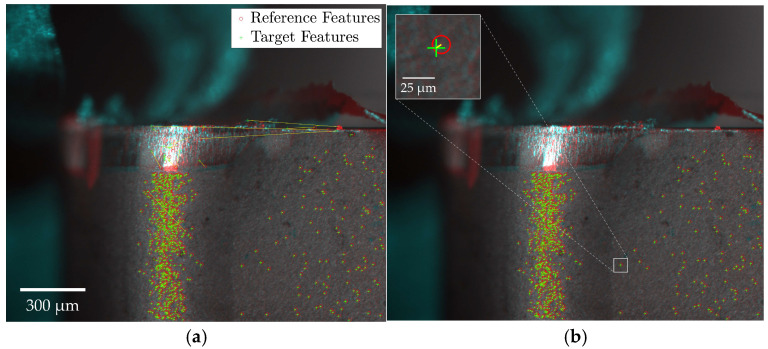
MV-TCM registration with adhered material formation using the T_3_ dataset, (*I_ref._* = I_4_) & (*I_tgt._* = I_5_), and employing the SIFT algorithm (*T_R_* = 0.6). Registration depicted using overlayed images (**a**) including all matched feature pairs and (**b**) following outlier removal using MSAC algorithm.

The registration process was shown to be insensitive to large surface artifacts, such as welded-on chips, depicted in Figure 8, independent of the feature detector-descriptor used. Few matched feature pairs were detected in these regions, as shown in Figure 8a, demonstrating the feature-based algorithms’ ability to prioritize distinctive, invariant features while disregarding transient anomalies. This ensures that formations like welded-on chips do not compromise registration reliability. The remaining false-positive matches were effectively removed, as depicted in Figure 8b.

**Figure 9:**
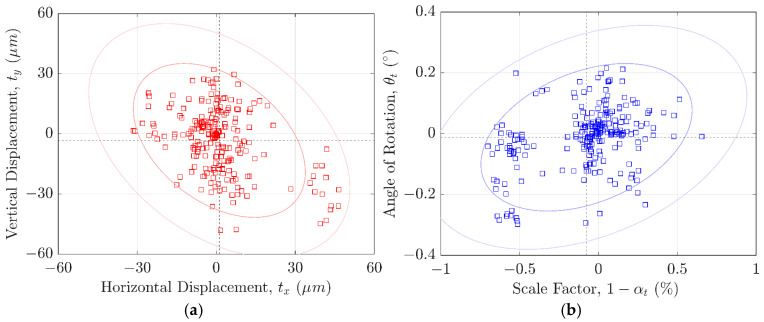
Similarity transformation coefficients extracted from successfully registered MV images illustrated as (**a**) Horizontal and vertical displacement (μm), (**b**) Scale factor (%) and angle of rotation (°). Solid lines represent confidence ellipses of 95.0% and 99.9%. Dashed lines represent coefficient mean.

The similarity transformation matrix coefficients obtained from successfully computed registrations were extracted and plotted, as shown in Figure 9. Horizontal and vertical displacements are presented in Figure 9a, while the rotation angles and scale factors are depicted in Figure 9b.

The horizontal displacement distribution was measured at 1.2 ± 13.3 μm and the vertical at −3.3 ± 15.7 μm, with all xy-displacements confined within ±50 μm. While these coefficients provided valuable insights into the system’s overall positional accuracy and repeatability, they were insufficient for assessing registration validity alone, as failed registrations occasionally exhibited displacement values within the expected range of successful ones. In contrast, the scale factor and angle of rotation proved to be more reliable indicators, with failed registrations showing significantly different values in one or both coefficients. The scale factor distribution for successful registrations was measured at −0.075 ± 0.27%, with minimum and maximum values of −0.701% and +0.655%, respectively. These extremes corresponded to approximate positional displacements along the MV axis of −11.3 μm and 10.4 μm, which, despite exceeding the prescribed DOF of 12 μm, did not introduce noticeable image blurring. The angle of rotation distribution measured −0.012 ± 0.100°, with minimum and maximum values of −0.298° and 0.216°, respectively.

#### 3.2.2. Utilizing the Mixed KAZE-SIFT Algorithm for Challenging Registrations

Challenges in the field of image registration are most often due to one or more of the following conditions: (1) images are captured from differing sensing modalities; (2) complex, nonlinear transformations are required to map the registration; and (3) a significant amount of content in the images have changed. While the first two conditions do not apply to MV-TCM registration, prolonging the time between the target and reference image may present challenges as the level of tool wear progresses. As a result, the registration framework is investigated and discussed further in Section 3.2.4.

Challenging registrations are often characterized by fewer correctly matched feature pairs, a corresponding increase in outlier pairs, inaccurately fitted transformations, or registration failures due to an insufficient number of detected inlier feature pairs. When assessing the failed registrations produced in Section 3.2.1, it was observed that a disproportionate number of the failed attempts corresponded to registrations between the initial unworn and the first worn tool image—coincidently where the onset of flank wear led to a significant increase in the overall image brightness. Of the 1135 registrations computed, 26 were unsuccessful, with 12/26 (46%) failures occurring between unworn and worn tool images. While registrations between unworn and worn images represented a minority, 60/1135 (5%), they accounted for nearly half of the failures, highlighting the particular challenge of registering these images. Similar difficulties were observed when inconsistent lighting affected image capture at other stages.

**Figure 10:**
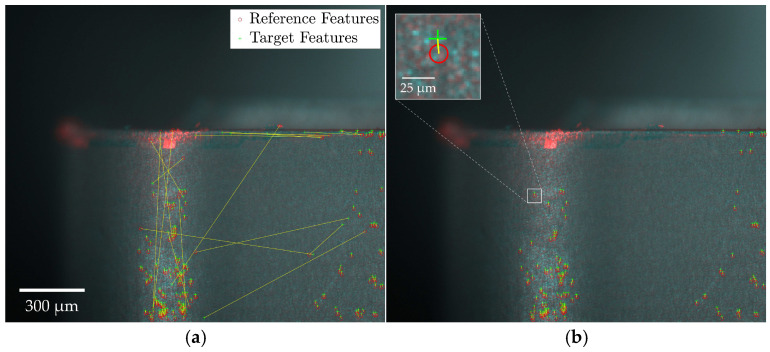
MV-TCM registration of unworn and worn tools using the T_5_ dataset, (*I_ref._* = I_0_) & (*I_tgt._* = I_1_) and employing the KAZE-SIFT algorithm (*T_R_* = 0.6). Registration depicted using overlayed images (**a**) including all matched feature pairs, and (**b**) following outlier removal using MSAC algorithm.

**Figure 11:**
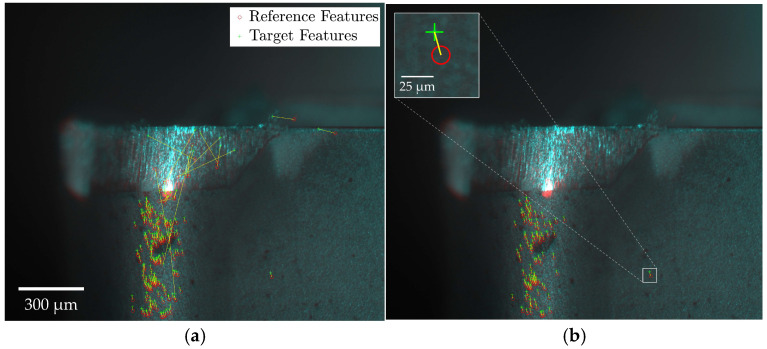
MV-TCM registration with compromised lighting using the T_4_ dataset, (*I_ref._* = I_23_) & (*I_tgt._* = I_24_) and employing KAZE-SIFT algorithm (*T_R_* = 0.6). Registration depicted using overlayed images (**a**) including all matched feature pairs, and (**b**) following outlier removal using MSAC algorithm.

The mixed KAZE-SIFT algorithm was applied to challenging conditions and demonstrated a significant increase in the number of detected inlier feature pairs compared to either algorithm used individually. During initial image registrations, the KAZE-SIFT algorithm produced a 30% increase in inlier feature pairs compared to KAZE alone, with this improvement rising to 54% across subsequent registrations. The following figures illustrate sample challenging registrations using the KAZE-SIFT algorithm, with Figure 10 presenting an initial registration and Figure 11 depicting a registration under compromised illumination conditions.

The increase in detected inlier feature pairs with the KAZE-SIFT algorithm enabled successful alignment across all challenging registrations. However, the number of inlier feature pairs remained relatively low and showed considerable variability between image sets, indicating that the approach may not fully resolve the underlying factors affecting these cases. This ongoing reduction in inlier features ultimately poses a challenge for the registration process, suggesting that more advanced feature detector-descriptor algorithms may be required to effectively address the impact of illumination variability.

#### 3.2.3. Effect of Tool Coating Variation on Registration Performance

Given the impact of illumination variability on registration performance and the range of tool coatings used in industrial applications, the effect of coating variation on the registration process was evaluated. Additional cutting tests were performed using three coating types: (1) uncoated (silver), (2) TiN-coated (gold), and (3) TiAlN-coated (black). Registrations were performed sequentially across all five feature detector-descriptors. Table 8 summarizes the registration performance for the KAZE algorithm (*T_R_* = 0.6).

**Figure 12:**
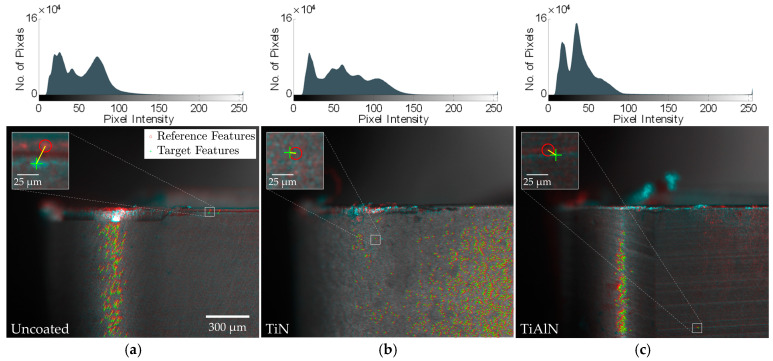
MV-TCM registration of various coated tools employing the SIFT algorithm (*T_R_* = 0.6), along with corresponding greyscale image histograms. Coating conditions include (**a**) uncoated, (**b**) TiN-coated, and (**c**) TiAlN-coated inserts.

The brighter TiN-coated inserts yielded nearly twice the number of detected features, matched pairs, and inlier pairs compared to the uncoated inserts, suggesting that higher surface illumination generally enhances feature detection. However, the darker TiAlN-coated inserts closely matched the uncoated results despite the shift in brightness, indicating that factors beyond image intensity, such as surface texture, may also influence registration efficacy. Representative registrations for each coating condition, along with their corresponding greyscale image histograms, are shown in Figure 12.

The distribution of inlier feature pairs across the tool surface exhibited distinct characteristics based on the specific coating condition examined. In the uncoated and TiAlN-coated conditions, inlier feature pairs were primarily concentrated in a narrow vertical strip between the primary and secondary flank faces, where illumination was most intensely reflected. In contrast, the TiN-coated inserts did not display this pronounced reflective band but instead provided sufficient illumination across the flank face, resulting in a more uniform distribution of inlier feature pairs throughout the tool surface.

While these findings indicate a complex interplay between illumination and surface texture, both of which significantly influence the overall performance of the feature-based registration process, they demonstrate that the evaluated feature detector-descriptor algorithms can still be effectively applied to cutting tools with a variety of coatings.

#### 3.2.4. Effect of Fixed vs. Sequential Framework on Registration Performance

Given the increasing difficulty in achieving successful image registrations as the time interval between reference and target images expands, as noted within the literature, the reference image selection strategy was analyzed. Registrations can follow either a fixed framework, where a single, unchanging reference image is used for all subsequent registrations, or a sequential framework, where the reference image is continuously updated. To evaluate the influence of these strategies on MV-TCM registration efficacy, both approaches were applied to the collected datasets, using the number of matched inlier feature pairs as an indicator of registration robustness.

**Figure 13:**
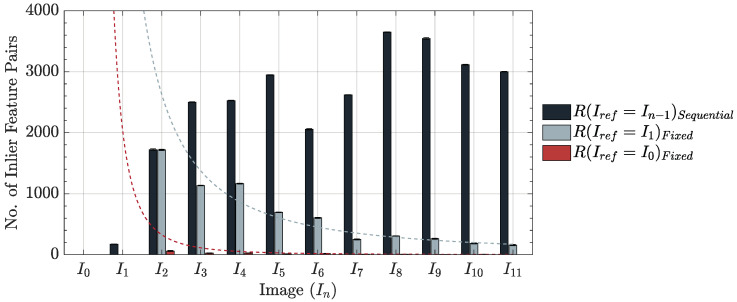
Detected inlier feature pairs using a sequential vs. fixed framework. T_2_ dataset utilized, and KAZE algorithm (*T_R_* = 0.6) employed. Datapoints represent average of *N* = 10 registrations. Error bars represent the maximum difference between trials. Dashed lines represent fitted trendlines.

The following three cases were examined: (1) sequential framework, utilizing the most recently registered target image ($I_{n-1}$) as the reference; (2) fixed framework, employing the initial worn image of the cutting tool ($I_{1}$) as the reference, and (3) fixed framework, using the initial unworn image of the cutting tool ($I_{0}$) as the reference. Registrations were performed using all five feature detector-descriptor algorithms. Figure 13 presents a comparative example between the fixed and sequential-based registration frameworks.

The number of inlier feature pairs detected under fixed registration frameworks followed a power-law decline, resulting in a significant increase in failed registrations compared to the sequential framework. This trend was consistent across all datasets, regardless of the feature detector-descriptor employed. The reduction was primarily attributed to textural changes on the tool surface caused by the machining process, with the decline shown to be proportional to the time elapsed between the target and reference images. The ($I_{ref}=I_{0}$) fixed framework similarly exhibited a power-law decline, with an order of magnitude fewer detected inlier feature pairs compared to the ($I_{ref}=I_{1}$) framework. This trend was consistent across all datasets and was directly attributable to the increased difficulty of registering images between unworn and worn cutting tools, as detailed in Section 3.2.2. The challenge was particularly impactful when employing algorithms like SIFT, SURF, BRISK, and ORB, which typically detected fewer inlier feature pairs, leading to a higher number of unsuccessful registrations.

In contrast, the number of inlier feature pairs detected under the sequential framework varied across registrations, showing no consistent trend due to the continuous selection of a new reference image for each instance. Despite these fluctuations, the sequential framework consistently yielded a higher number of inlier feature pairs compared to the fixed framework, resulting in fewer registration errors. This pattern was observed regardless of the feature detector-descriptor algorithm used.

**Figure 14:**
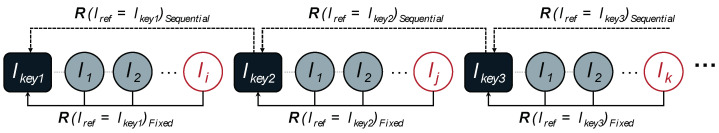
Proposed hybrid registration sequence for drift mitigation.

While the sequential framework detects more inlier feature pairs, it also introduces the risk of registration drift due to the accumulation of transformation errors over successive registrations. This drift increases with the number of sequential registrations and associated errors and may also be influenced by the complexity of the transformations. In this study, the *estgeotform2d* function in MATLAB R2022b, using its default *MaxDistance* of 1.5 pixels, indicated a theoretical maximum transformation error of 1.05 µm per registration. Despite these potential concerns, no registration drift was observed across the 12 MV datasets, each consisting of 10 to 36 cutting tool images. To further mitigate the risk of drift, a hybrid framework that combines sequential and fixed reference strategies could be considered. An example registration framework is provided in Figure 14.

The framework operates by selecting key frame images at fixed intervals (N) or adaptive intervals ($N_{i}=f(n_{inliers}$)), where key frame images are registered sequentially to one another, and intermediate images are registered to the preceding, fixed key frame. This approach reduces registration drift by a factor of $\bar{N}$; however, the implementation and overall effectiveness of this approach warrant further investigation.

#### 3.2.5. Automated MV-TCM Registration Algorithm

An automated, feature-based registration algorithm was developed, informed by the following key observations drawn from the preceding chapters:

- The registration algorithm comparative case study revealed the following rankings:
  - Registration accuracy: KAZE > SIFT > ORB > BRISK > SURF
  - Registration time: ORB > SURF > BRISK > SIFT > KAZE
  - ITD feature ratio: KAZE > SIFT > SURF > BRISK > ORB
- The scale factor and angle of rotation were identified as being the most reliable similarity transformation coefficients for distinguishing between successful and failed registrations.
- Challenging registrations were mostly influenced by the following:
  - Abrupt changes in the illumination, primarily between the unworn and first worn tool images, however, were mitigated through the implementation of a hybrid KAZE-SIFT feature detector-descriptor algorithm.
  - The gap in time between the reference and target images, however, was mitigated through the use of a sequential-based registration framework.

**Figure 15:**
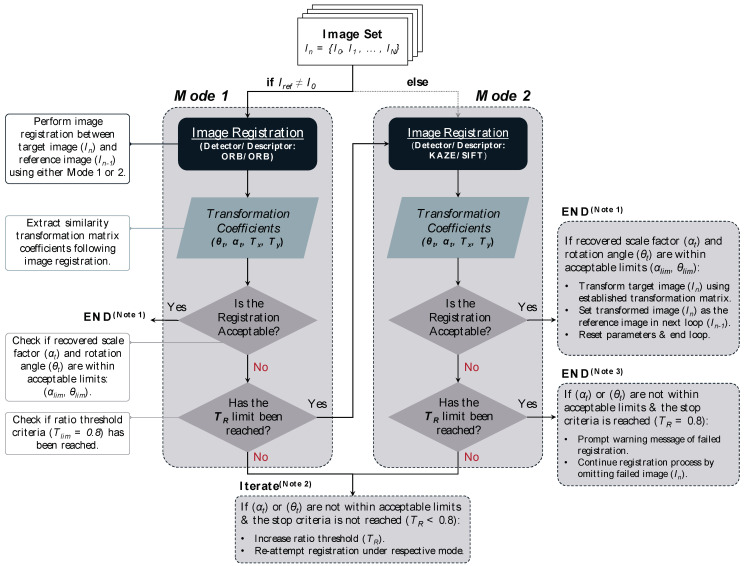
Flowchart presenting the automated, feature-based MV-TCM registration algorithm.

The automated MV-TCM registration algorithm flowchart is presented in Figure 15. The algorithm balances efficiency and accuracy through a dual-mode registration strategy. Mode 1, the default mode, employs the ORB algorithm, selected for its superior computational speed and sufficient registration accuracy, as demonstrated in Section 3.2.1. The computational efficiency of ORB enables multiple registration attempts within a shorter timeframe, improving the effectiveness of the ratio threshold refinement process. In contrast, Mode 2 utilizes the mixed KAZE-SIFT algorithm, selected for its ability to increase the number of detected inlier feature pairs, as established in Section 3.2.2. This mode is employed under challenging cases where accuracy is prioritized over computational speed, such as initial registrations between unworn and worn tool images or when Mode 1 fails to achieve successful registration despite ratio threshold refinement.

The algorithm employs a sequential framework, updating the reference image to the most recently registered target image due to its ability to detect a greater number of inlier feature pairs and achieve a higher registration success rate compared to fixed reference methods, as shown in Section 3.2.4. Following each registration, the scale factor and angle of rotation transformation coefficients are extracted to evaluate registration validity. This validation process is automated by cross-referencing these coefficients with their critical threshold limits, which were established using Equations (6) and (7), informed by the transformation coefficients of successful registrations presented in Section 3.2.1.

(6) $$\left|\theta_{t}\right|\leq \theta_{lim}=0.4{}^{\circ},$$

(7) $$\left|1-\alpha_{t}\right|\leq \alpha_{lim}=1\%,$$

When the transformation coefficients fail to meet the specified registration accuracy criteria, the algorithm iteratively adjusts the ratio threshold to identify the value necessary for accurate registration. If the threshold limit $\left(T_{lim}=0.8\right)$ is reached without success in Mode 1, the algorithm reattempts the registration in Mode 2, following the same refinement process. If the registration remains unsuccessful and the ratio threshold limit is reached once again, the registration is deemed unsuccessful. Following this point, the algorithm excludes the failed image and proceeds with the subsequent registrations.

Previously acquired MV images were used to assess the performance of the registration algorithm. A summary of the results is provided in Table 9.

The registration algorithm successfully registered all previously collected MV dataset images, achieving an average computational time of 1.3 s per registration. While further enhancements could be made by incorporating advanced feature-detector descriptor algorithms and customizing threshold limits based on the confidence ellipses of the transformation coefficients rather than the uniform limits currently used, the presented algorithm effectively facilitates the automated registration of MV-TCM images.

### 3.3. Registration-Based Edge Detection Strategy for TCM

#### 3.3.1. Reference Line Detection Strategies

Flank wear measurements are obtained by calculating the perpendicular distance between two parallel lines: one anchored along the tool edge and the other representing the tool wear line, which is defined based on the wear morphology. While the placement of the wear line may vary depending on operator interpretation, the consistent and precise detection of the reference line remains critical for obtaining reliable wear measurements. This challenge becomes pronounced when the tool edge is compromised, either by material adhesion, which can result in the overestimation of wear due to the misclassification of BUE or welded-on material as wear, or by edge chipping, which can lead to the underestimation of wear by failing to detect wear pixels in the damaged regions.

The current standard for reference line detection employs the Hough transform, which has been shown to be effective in identifying straight lines dispersed throughout an image and is particularly functional for the application of tool segmentation and wear classification [16]. However, its application for flank wear measurement has yet to be fully explored, particularly under increasing levels of edge deterioration.

This study develops an alternative approach, leveraging spatial registration of MV images followed by edge detection on the initial unworn tool image to infer edge positions. This method ensures consistent reference lines across the entire image set, reducing processing complexity and minimizing the risk of erroneous reference line detection. To evaluate the effectiveness of each reference line detection strategy, wear assessments were extracted from MV images under three conditions: (1) the Hough transform strategy, (2) the registration-based strategy, and (3) a control condition without edge detection. Maximum flank wear (*Vb_max_*) measurements were obtained using a standard image processing workflow, including Gaussian smoothing, greyscale conversion, image binarization, morphological operations, and pixel-to-distance conversion. All processing parameters were kept constant to ensure consistency across tests.

**Figure 16:**
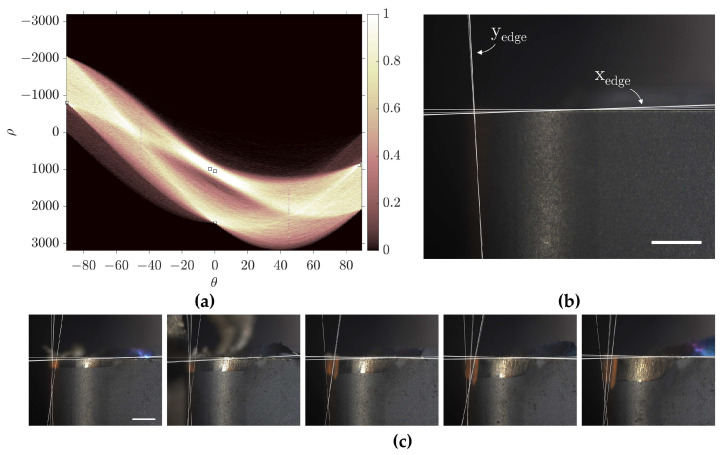
Hough transform strategy for reference line detection. Process consists of (**a**) application of Hough transform and identification of Hough peaks, (**b**) extrapolation of line segments to infer *x_edge_* & *y_edge_*, and (**c**) Reiteration across all other images in image set.

The Hough transform strategy begins by applying the Sobel edge operator to identify potential edges, which are then mapped to Hough space using the *hough* MATLAB function. Peaks in the Hough space, corresponding to the accumulation of intersecting sinusoidal curves, represent straight line segments that are detected using the *houghpeaks* function. The Hough transform generates multiple inferred tool edge lines, from which the edge line best representing the true cutting tool reference is selected for further analysis. Figure 16 illustrates the Hough transform strategy for reference line detection prior to the filtering process.

**Figure 17:**
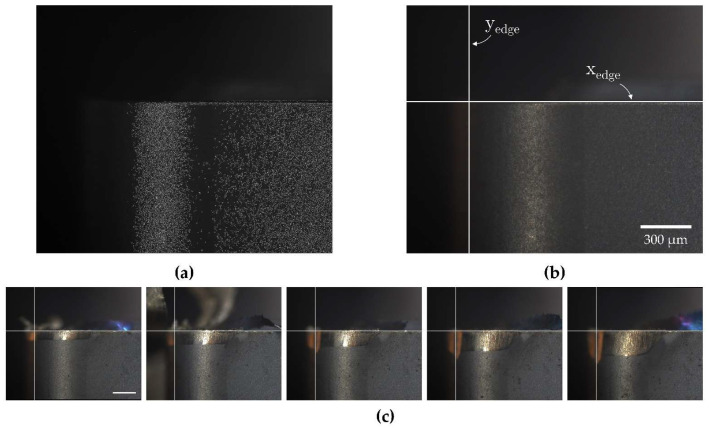
Proposed image registration-based strategy for reference line detection. Following registration of image set, process consists of (**a**) Canny edge operator on (*I*_0_), (**b**) bounding box outline to infer *x_edge_* & *y_edge_*, and (**c**) application of edge lines across dataset.

The registration strategy applies the automated registration algorithm, outlined in Section 3.2.5, followed by a Canny edge operator to the initial unworn tool image (*I₀*) to outline the contour of the cutting tool. A bounding box is fitted around the detected pixels to define the horizontal and vertical reference edge lines, which remain fixed throughout subsequent image processing. In cases where only one edge is present, as described in Section 4, a single straight line replaces the bounding box. The registration strategy is presented in Figure 17.

#### 3.3.2. Application of Registration-Based Reference Line Detection Strategy for TCM

**Figure 18:**
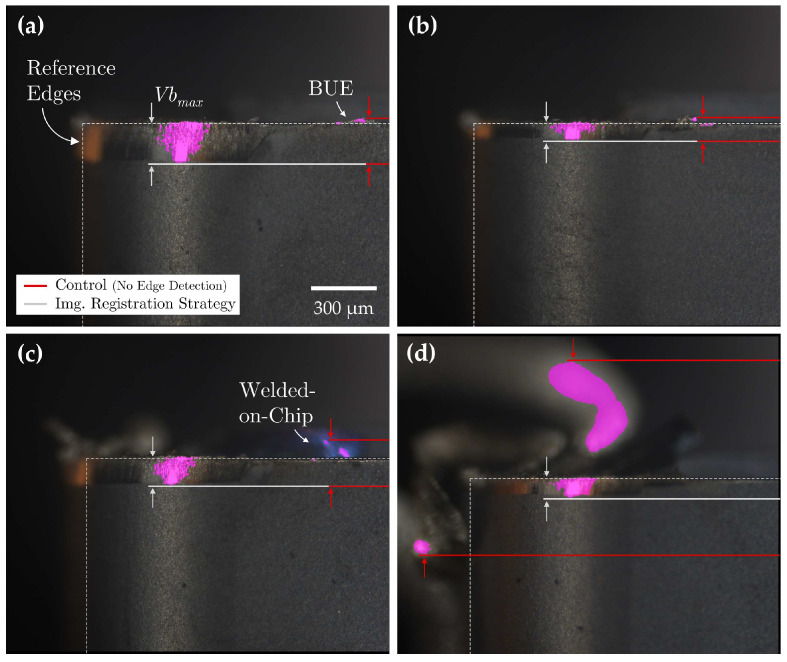
Flank wear (*Vb_max_*) assessment with and without utilization of the registration-based reference line detection strategy. The areas highlighted in pink correspond to the binary mask generated through image processing for wear assessment. Cases utilize dataset images with varying levels of adhered material formation: (**a**) Mild BUE, (**b**) Mild-Moderate BUE, (**c**) Moderate chip, and (**d**) Severe chip formation.

Figure 18 provides a visual comparison of flank wear assessments obtained using the registration-based reference line detection strategy versus the control condition. To reduce visual complexity, reference lines detected through the Hough transform strategy have been omitted from the figure. A comprehensive summary of the quantitative flank wear assessment results across all three conditions is presented in Table 10.

The accuracy in the assessment of flank wear measurements through the applications of a standard, unchanging sequence of image processing declined proportionally with increasing levels of BUE and welded-on chip formations. While regions of erroneously segmented tool wear regions located away from the wear region could be corrected by fine-tuning processing parameters, correcting the misclassified regions of substrate material smeared ahead of the reference line and in direct contact with the wear region proved infeasible without compromising the integrity of the wear segmentation.

The integration of both reference line detection strategies significantly improved the precision of flank wear measurements, particularly under conditions of adhered material formation. Measurement errors were reduced to 15.0% using the Hough transform method and further minimized to 2.5% with the registration-based strategy. While the Hough transform consistently identified reference lines regardless of the level of adhered material, its accuracy was highly dependent on the edge detection algorithm and threshold parameters employed. Moreover, the Hough transform produced multiple inferred reference lines per tool edge, each exhibiting slight variations in angle and position, requiring additional processing to select the most accurate edge representation. Conversely, the registration-based strategy demonstrated superior accuracy in detecting true edge positions by confining edge detection to the initial unworn tool image. The high-level registration accuracy across images ensured that reference lines remained consistent throughout the dataset, resulting in significantly improved measurement accuracy.

## 4. Case Study: MV-TCM Application Within a CNC Milling Center

### 4.1. MV Setup, Data Collection, & Image Registration

**Figure 19:**
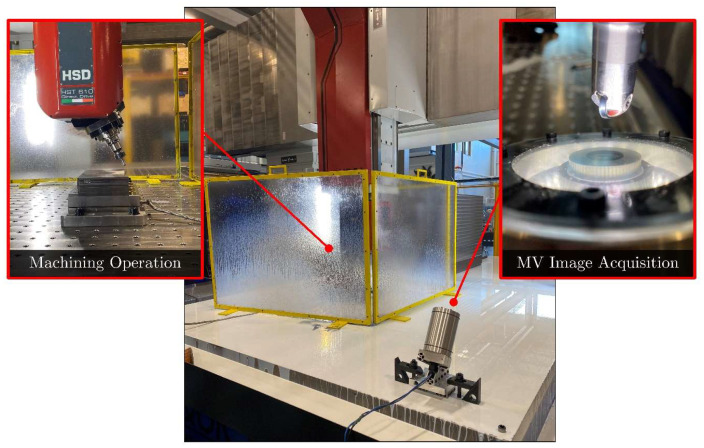
MV-TCM experimental setup for CNC milling center.

To assess the broader applicability and effectiveness of the MV-TCM strategy, the MV system was integrated into the QUICKMILL Eliminator HD 5A CNC milling center. The experimental setup is depicted in Figure 19.

**Figure 20:**
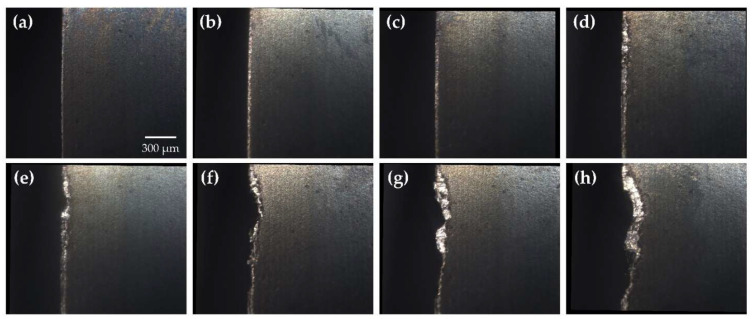
Tool wear images captured from the MV system from the M_6_ dataset at cutting passes: (**a**) P = 0; (**b**) P = 860; (**c**) P = 1635; (**d**) P = 3280; (**e**) P = 3750; (**f**) P = 4220; (**g**) P = 4455; and (**h**) P = 5160.

Tool life-cutting tests were conducted as outlined in Section 2.3.2. Following the collection of tool wear images, the automated registration algorithm was employed to spatially align all images. Registered cutting tool images are presented in Figure 20.

The tool wear images were shown to be of high quality, with the wear regions clearly detailed and distinguishable from unworn areas. Minor misalignment of the MV system relative to the cutting tool, along with the curvature of the milling insert, introduced an illumination gradient across the tool surface and slight blurring in areas distant from the focal point. Additional limitations were observed in the experimental setup as residual coolant stains were presented on the cutting tool despite attempts to clean the surface using compressed air. As a result, manual cleaning was required at times. Despite these imperfections, the image quality was considered acceptable.

The tool positioning remained accurate throughout cutting tests, ensuring positional consistency across collected images. However, positioning precision was more variable than in previous turning experiments, likely due to the increased travel path and degree of freedom engaged during the repositioning of the CNC machine tool. As a result, the registration algorithm did not meet the original transformation threshold limits specified in Section 3.2.5. Consequently, the angle of rotation and scale factor limits were adjusted to meet the new conditions for successful registration, after which all images were registered successfully. Future work may require the use of image transformations with greater DOF to more accurately capture the spatial transformations encountered in such conditions.

### 4.2. Tool Wear Assessment Utilizing the Registration-Based Reference Line Detection Strategy

In contrast to the turning experiments, where tool wear was primarily characterized by adhered material formation, the milling tests resulted in progressive chipping of the tool edge. Although this wear mechanism is generally undesirable in standard machining, it provides a valuable opportunity to evaluate the effectiveness of each reference line detection strategy in assessing tool wear under the opposing condition of edge degradation.

**Figure 21:**
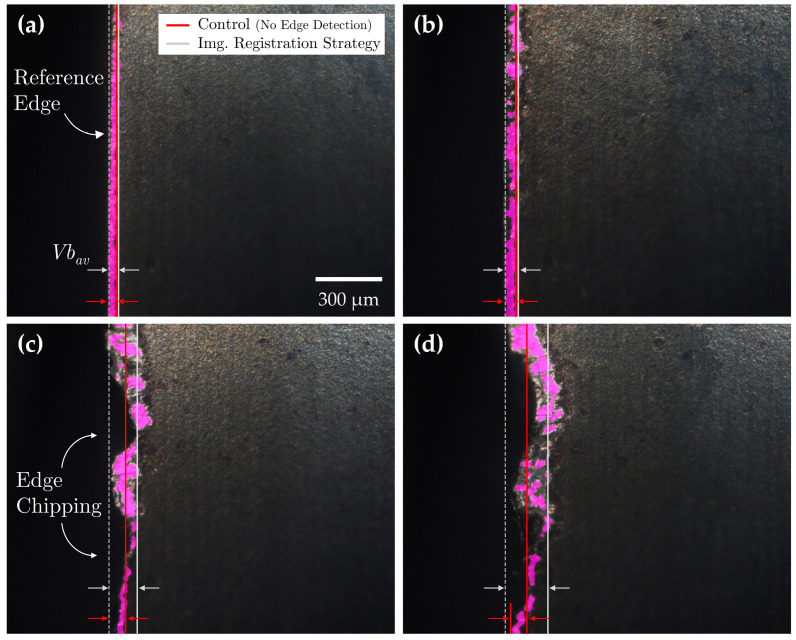
Flank wear (*Vb_av_*) assessment with and without utilization of the registration-based reference line detection strategy. The areas highlighted in pink correspond to the binary mask generated through image processing for wear assessment. Dataset utilized images featuring varying levels of edge chipping: (**a**) No chipping, (**b**) Mild chipping, (**c**) Moderate chipping, and (**d**) Severe chipping.

Tool wear measurements were extracted from MV images using the same three conditions: (1) the Hough transform strategy, (2) the registration-based strategy, and (3) a control condition without edge detection. Average flank wear measurements (*Vb_av_*) were obtained using a similar image processing workflow with two key modifications: adaptive thresholding was used for image binarization to address illumination gradients, and images were segmented into 100 µm sections to compute local (*Vb_max_*) measurements based on the differences between the left and right most detected pixels. Tool wear measurements were computed by evaluating the mean of the local (*Vb_max_*) measurements across each segment. Under edge detection methods, the leftmost pixel would be taken at the corresponding reference line. Figure 21 provides a comparison of flank wear assessments obtained using the registration-based reference line detection strategy versus the control condition. Table 11 summarizes the flank wear assessment results across all three conditions.

The accuracy of flank wear measurements without a reference line detection strategy showed a proportional decline as the severity of edge chipping increased unlike the turning experiment, where locally isolated, erroneously segmented adhered material could most often be corrected by adjusting thresholding parameters, the complete absence of material in areas affected by chipping rendered such corrections ineffective, making precise flank wear assessments under these conditions unattainable.

Although the Hough transform reference line detection strategy improved flank wear assessment accuracy in the absence or presence of mild edge chipping, it produced inadequate results under moderate and severe chipping conditions. The failure to accurately identify tool edge positions in these cases was not due to the limitations of the algorithm itself but rather the inappropriate application of the method under conditions where the tool edge had deteriorated beyond visual identification. In contrast, the registration-based reference line detection strategy significantly improved flank wear assessment accuracy across all levels of edge chipping, reducing assessment errors to 4.5% relative to true flank wear measurements. The following case study highlights the versatility of the MV system across diverse machining environments while demonstrating the importance of the registration-based reference line detection strategy in simplifying the edge detection process and enhancing tool wear assessments.

## 5. Conclusions

MV systems for TCM have demonstrated significant potential for reducing manufacturing costs and expediting tool wear testing in research laboratories. However, their practical implementation often falls short of expectations, primarily due to challenges in accurately detecting the tool edge line, which serves as the reference mark for wear assessment. Existing methods rely on detecting the onset of tool wear to estimate the edge position or iteratively applying the straight-line Hough transform to each image; however, these approaches are inherently sensitive to irregularities in the tool edge caused by material adhesion and edge chipping formed during machining operations. More importantly, they do not adequately address positional variability between the MV system and the cutting tool—the underlying factor leading to the inaccuracies in edge detection.

This study details the development and implementation of an MV system integrated with a registration-based strategy for reference line detection in TCM. The MV system was designed to address common limitations in existing designs, including susceptibility to vibrations, swarf, coolant, and lighting variations. The influence of the swarf was minimized by developing a protective enclosure to shield the sensing equipment, while coolant exposure was reduced by sealing the enclosure and incorporating an air jet nozzle for tool cleaning. Lighting variations were reduced by integrating the illumination system within the MV housing, and the influence of vibrations was minimized by mounting the MV system on the CNC machine headstock rather than the back panel. Custom 3D-printed components were also incorporated into the housing to provide additional support for the imaging equipment.

The resulting MV system delivered image quality comparable to conventional digital microscopes, exhibited robustness to the machining environment, and reduced image acquisition time by up to 85% relative to standard TCM practices. Limitations were observed during instances where the air jet cleaner was unable to fully remove coolant stains from the tool surface, despite its general effectiveness in dislodging larger welded-on chips and removing debris. Future designs should focus on improving tool cleaning mechanisms to improve image quality.

To develop the registration-based strategy for reference line detection, an automated feature-based registration algorithm was designed based on the following key findings:

- The comparative case study of feature detector descriptor algorithms identified SIFT, KAZE, and ORB as the most suitable for MV-TCM applications. Among these, KAZE demonstrated the lowest rate of failed registrations, while ORB offered the highest computational efficiency, and SIFT presented a balance of the two. Additionally, the analysis established that transformation coefficients, specifically the scale factor and angle of rotation, served as effective metrics for automatically evaluating registration success.
- Challenging registrations were primarily influenced by abrupt changes in illumination, particularly between unworn and first-worn images, as well as extended time intervals between reference and target images. These challenges were mitigated by employing a hybrid KAZE-SIFT feature detector-descriptor algorithm and a sequential registration framework, respectively.

The registration algorithm demonstrated both effectiveness and efficiency, successfully registering all MV images with an average time of 1.3 s per image. It performed reliably across various cutting tool geometries and coating types, ensuring robustness under diverse machining conditions. Despite its success, improvements could be made by incorporating more advanced feature detector-descriptor algorithms and implementing a more rigorous screening process to validate registrations. Additionally, while the algorithm was effective for TiN and TiAlN-coated tools, an unexpected relationship was observed between the number and spatial distribution of feature pairs detected during registration. Future work should focus on further validating the approach across additional coating conditions and investigating the influence of tool coating, surface texture, and image brightness on the performance of feature-based registration methods.

The registration-based strategy for reference line detection provided a more precise and consistent assessment of tool edge positions compared to the standard straight-line Hough transform commonly used in the literature. In turning tool life tests, where material adhesion was prominent, the Hough transform produced a flank wear error of 15% when compared to the manual wear measurements taken from the KEYENCE digital microscope. Comparatively, this error was reduced to 2.5% using the registration-based approach. In milling tool life tests, which involved progressive edge chipping, the Hough transform failed under moderate to severe chipping, while the registration-based method remained unaffected, achieving an average flank wear error of 4.5%.

The limitations of the Hough transform stemmed less from the algorithm itself and more from its independent application to each image, failing to address positional variability. The registration-based strategy addressed this issue by spatially aligning tool wear images, simplifying edge detection, and enabling more consistent and reliable wear assessments. Current limitations of the registration-based approach remain to be conditions of abrupt illumination changes, in which the number of detected inlier feature pairs remained consistently low and highly variable despite mitigation efforts. Future studies could explore additional feature detector-descriptor algorithms to overcome these challenges. In addition, while similarity transformations proved effective in this study, incorporating transformations with higher degrees of freedom could enhance alignment accuracy by capturing more complex spatial transformations. This improvement would allow for more rigorous registration criteria and reduce the likelihood of incorrectly classifying failed registrations as successful. Furthermore, registration drift was not observed in this study; however, its potential impact was acknowledged, and a hybrid registration framework combining sequential and fixed-based strategies was proposed. Future efforts aimed at detecting registration drift would further enhance this approach, particularly in applications involving a significantly larger number of images. Lastly, while this work validated the approach for simple straight tool edges, the framework is expected to be adaptable to cutting tools with more complex edge profiles, warranting further investigation.

The MV system and registration-based reference line detection strategy developed in this study enabled the acquisition of high-quality tool wear images and ensured more consistent and accurate assessments of tool wear. These advancements expand potential applications of MV systems in tool wear monitoring processes and further promote data-driven condition-based maintenance strategies in TCM.

## Figures and Tables

**Table 1:** Feature detector-descriptor algorithms used for MV-TCM registration.

Case	Registration Method		MATLAB-R2022b	Detector		* Descriptor		
	Ref.	Detector- Descriptor	Function	Parameter	Value	Invariance		Binary
						Scale	Rotation	
1	[25]	SIFT (blob)	detectSIFTFeatures	ContrastThreshold	0.0133	✓	✓	✗
				EdgeThreshold	10.0			
				NumLayersInOctave	3			
				Sigma	1.6			
2	[26]	SURF (blob)	detectSURFFeatures	MetricThreshold	1000	✓	✓	✗
				NumOctaves	3			
				NumScaleLevels	4			
3	[27]	KAZE (blob)	detectKAZEFeatures	Threshold	0.0001	✓	✓	✗
				NumOctaves	3			
				NumScaleLevels	4			
4	[28]	BRISK (corner)	detectBRISKFeatures	MinContrast	0.2	✓	✓	✓
				MinQuality	0.1			
				NumOctaves	4			
5	[29]	ORB (corner)	detectORBFeatures	ScaleFactor	1.2	✗	✓	✓
				NumLevels	8			

* Check marks indicate the presence of scale or rotation invariance and binary representation, while crosses denote their absence.

**Table 2:** Turning test conditions, machining parameters, and MV datasets developed for tool life-cutting tests. A constant depth of cut of 0.5 mm was used throughout testing.

Test Condition	Cutting Speed, *V_c_*	Feed Rate, *f*	MV Dataset
	(m/min)	(mm/rev)	
C_1_	125	0.075	T_7_, T_9_
C_2_	125	0.100	T_4_, T_11_
C_3_	150	0.075	T_1_, T_5_
C_4_	150	0.100	T_2_, T_8_
C_5_	175	0.075	T_10_, T_12_
C_6_	175	0.100	T_3_, T_6_

**Table 3:** Milling test conditions, machining parameters, and MV datasets developed for tool life tests. A constant axial and radial depth of cut of 0.406 mm and feed rate of 0.19 mm/tooth were used.

Test Condition	* Workpiece Material	Spindle Speed	MV Dataset
		(RPM)	
C_1_	Finkl P20 MD	10,000	M_1_, M_2_
C_2_	Finkl P20 MD	12,000	M_3_, M_4_
C_3_	Daido PX5	10,000	M_5_, M_6_
C_4_	Daido PX5	12,000	M_7_, M_8_

* Workpiece material provided by Tycos Tool & Die (Concord, ON, Canada).

**Table 4:** TCM task breakdown comparison between the standard and MV-TCM process.

Method	Setup/Removal	Image Capture
Standard-TCM	Remove and reinstall the cutting tool insert into the CNC machine’s tool holder. Moving between the CNC machine and the digital microscope inspection station.	Aligning the cutting tool under the digital microscope, executing focus stacking, and capturing the image. Manually clearing debris from the cutting tool as necessary.
MV-TCM	The CNC controller repositions the cutting tool to the machine vision system for image acquisition and subsequently returns it to the workpiece for machining.	Capturing four consecutive images at one-second intervals. Utilizing an air jet to clear welded-on chips, BUE, or other debris as necessary.

**Table 5:** Time study analysis between standard and MV-TCM systems. Test conditions correspond to cutting tests: T_7_, T_4_, T_1_, T_2_, T_10_, and T_3_ respectively. Time results are presented in minutes.

	Standard-TCM				MV-TCM				
Test Cond.	1. Machining	2. Setup/Removal	3. Image Capture	Total Time	1. Machining	2. Setup/Removal	3. Image Capture	Total Time	* Δ (%)
C1	13 (9%)	50 (36%)	75 (54%)	138	13 (40%)	10 (30%)	9 (29%)	32	−85%
C2	10 (10%)	39 (37%)	56 (53%)	106	10 (38%)	9 (33%)	8 (30%)	27	−82%
C3	7 (9%)	30 (39%)	41 (53%)	78	7 (36%)	6 (34%)	6 (30%)	19	−83%
C4	4 (6%)	24 (35%)	40 (59%)	68	4 (28%)	5 (34%)	6 (38%)	15	−84%
C5	3 (7%)	17 (35%)	28 (58%)	48	3 (33%)	4 (37%)	3 (30%)	10	−84%
C6	3 (7%)	15 (38%)	22 (55%)	40	3 (30%)	3 (35%)	3 (35%)	9	−83%

* Machining time excluded in the computation of the percentage change.

**Table 6:** Registration performance comparison results between various feature detector-descriptors.

Case	Reg. Method	Time	Number of Features				Results
	Detector- Descriptor	Per Image (s)	Reference Image	Target Image	Matched Features	Inlier Features	Registration Error # (%)
1	SIFT	2.90	1317	1424	257	225	2 (0.88%)
2	SURF	0.91	783	853	73	43	14 (6.17%)
3	KAZE	9.89	11,173	11,737	2234	1909	1 (0.44%)
4	BRISK	2.55	7156	7335	311	287	6 (2.64%)
5	ORB	0.50	6018	6306	259	204	3 (1.32%)

**Table 7:** Comparative performance summary of the feature detector-descriptor algorithms.

Case	Detector- Descriptor	Overall Accuracy	Overall Efficiency
1	SIFT	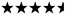	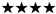
2	SURF	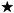	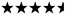
3	KAZE	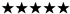	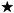
4	BRISK	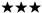	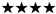
5	ORB	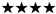	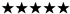

**Table 8:** Registration performance comparison for uncoated, TiN, and TiAlN-coated conditions.

	Tool Coating	Number of Features				
		Reference Image	Target Image	Matched Features	Inlier Features	ITD Ratio
1	Uncoated	9209	10,045	2171	1973	1:4
2	TiN-Coated	19,846	22,978	4136	3898	1:5
3	TiAlN-Coated	8679	9518	2055	1919	1:4

**Table 9:** Registration performance utilizing automated, feature-based MV-TCM algorithm.

Dataset/ (No. of Img.)		Time per Image (s)	Dataset/ (No. of Img.)		Time per Image (s)	Dataset/ (No. of Img.)		Time per Image (s)	Registration Error # (%)
T_1_	(19)	0.76	T_5_	(21)	1.24	T_9_ *	(36)	1.34	All images registered successfully
T_2_	(17)	0.81	T_6_	(11)	1.65	T_10_	(12)	1.30	
T_3_	(10)	1.65	T_7_	(31)	1.24	T_11_ *	(27)	1.37	
T_4_	(27)	1.13	T_8_	(16)	1.28	T_12_	(12)	1.68	

* *T_R_* threshold re-estimated during registration process.

**Table 10:** Comparative flank wear (*Vb_max_*) assessments utilizing the three edge detection strategies. Bolded entries indicate wear assessment values most closely aligned with true measured readings.

Case	Dataset (Pass #)	Description (Adhered Material)	*Vb_max_* Measurement	Control (No Edge Detection)	Hough Transform Strategy	Image Registration Strategy
			(µm)	(µm) Error	(µm) Error	(µm) Error
1	T_7_ (9)	Mild BUE	171.55	196.65 (14.6%)	188.85 (10.1%)	**177.13 (3.3%)**
2	T_7_ (1)	Mild-Moderate BUE	76.01	101.81 (33.9%)	87.80 (15.5%)	**75.31 (0.9%)**
3	T_3_ (2)	Moderate Chip	120.64	202.23 (67.6%)	134.49 (11.5%)	**123.43 (2.3%)**
4	T_2_ (1)	Severe Chip	78.80	* 857.04 (987.6%)	96.86 (22.9%)	**81.59 (3.5%)**

* Flank wear segmentation could not be improved by adjusting thresholding parameters.

**Table 11:** Comparative flank wear (*Vb_av_*) assessments utilizing the three edge detection strategies. Bolded entries indicate wear assessment values most closely aligned with true measured readings.

Case	Dataset (Pass #)	Description (Tool Chipping)	*Vb_av_* Measurement	Control (No Edge Detection)	Hough Transform Strategy	Image Registration Strategy
			(µm)	(µm) Error	(µm) Error	(µm) Error
1	M_6_ (860)	None	34.87	29.23 (16.2%)	32.08 (8.0%)	35.63 (2.2%)
2	M_6_ (3280)	Mild	51.60	41.90 (18.8%)	55.09 (6.8%)	49.20 (4.7%)
3	M_6_ (4455)	Moderate	108.79	60.42 (44.5%)	* 20.92 (80.8%)	102.83 (5.5%)
4	M_6_ (5160)	Severe	163.88	77.85 (52.5%)	* 66.95 (59.1%)	154.56 (5.7%)

* Inaccurate edge detection due to significant chipping of the cutting tool edge.

## Data Availability

The raw data supporting the conclusions of this article will be made available by the authors upon request.
